# The combination of high sensitivity troponin T and copeptin facilitates early rule-out of ACS: a prospective observational study

**DOI:** 10.1186/1471-2261-13-42

**Published:** 2013-06-18

**Authors:** Johan Thelin, Catharina Borna, David Erlinge, Bertil Öhlin

**Affiliations:** 1 Department of Clinical Sciences at Lund, Lund University, Lund, Sweden; 2 Skånes Universitetssjukhus, Akutmottagningen, Klinikgatan 15, 221 85, Lund, Sweden; 3 Skånes Universitetssjukhus, Kranskärlskliniken, 221 85, Lund, Sweden

**Keywords:** Copeptin, High sensitivity troponin, Acute coronary syndrome, Emergency department

## Abstract

**Background:**

The combination of the new high sensitivity troponin T (hsTnT) assays and copeptin, a biomarker of endogenous stress, has been suggested to have the potential of early rule-out of acute coronary syndrome (ACS). The aim of this study was to examine the ability of this combination to rule out ACS in patients presenting with chest pain and to compare the diagnostic performance to hsTnT alone.

**Method:**

In this prospective observational study, patients with chest pain admitted for observation were consecutively included. Patients presenting with ST elevation were excluded. Copeptin and hsTnT were analyzed at admission and hsTnT was thereafter determined approximately every 3^rd^ hour as long as clinically indicated. The follow-up period was 60 days. A combined primary endpoint of ACS, non-elective percutanous coronary intervention, non-elective coronary artery bypass surgery and death of all causes was used.

**Results:**

478 patients were included. 107 (22%) patients were diagnosed with ACS during hospital stay. 70 (14%) had non-ST-segment elevation myocardial infarction (NSTEMI) and 37 (8%) had unstable angina pectoris (UAP).

The combination of hsTnT >14 ng/L or copeptin ≥14 pmol/L at admission identified ACS with a higher sensitivity than hsTnT alone: 0.83 (95% confidence interval (CI): 0.74-0.89) versus 0.69 (95% CI: 0.59-0.77), p <0.001. Negative predictive values (NPV) 91% (95% CI: 86-94) versus 89% (95% CI: 84-92). A repeated hsTnT analyzed 3-4 hours after admission resulted in a sensitivity of: 0.77 (95% CI: 0.65-0.86), p =0.031 for comparison with the combination analyzed at admission.

**Conclusions:**

In patients presenting with chest pain admitted for observation, the combination of hsTnT and copeptin analyzed at admission had a significantly higher sensitivity to diagnose ACS than hsTnT alone. We report a sensitivity of 83% and a NPV of 91% for the combination of hsTnT and copeptin and we conclude that biomarkers alone are not sufficient to rule out ACS. However, the combination of hsTnT and copeptin seems to have a significantly higher sensitivity to identify ACS than a repeated hsTnT test, and thus enables an earlier risk stratification of chest pain patients. This can be time-saving and beneficial for the individual patient by contributing to early decisions on treatment, need of further assessment and level of care.

## Background

The leading symptom of an acute coronary syndrome (ACS) is typically chest pain. Since ACS is associated with a significant mortality and morbidity, correct diagnosis is of great importance [1]. Chest pain is a frequent symptom in medical emergency departments and distinguishing patients with ACS within the chest pain group is a diagnostic challenge. Patients with suspected ACS account for a substantial proportion of all acute medical hospitalizations and accordingly cause large costs for the health care system and inconvenience for the individual patient [2]. Cardiac troponins and electrocardiography (ECG) in combination with the medical history and physical examination are at present the diagnostic cornerstones. In recent years, high sensitivity troponin (hsTn) tests have been introduced [3,4], and it has been suggested that a rapid rule-out protocol with a second sample of hsTn after 3 hours is sufficient [1].

In the ideal scenario, ACS could be ruled out with sufficient accuracy already at presentation. A copeptin sample in combination with a cardiac troponin test has been suggested for such early rule-out [5,6].

Cardiac troponins are released after myocardial cell disintegration and are markers of cell necrosis. This may be the reason for the relative weakness in diagnostic performance in patients presenting early after chest pain onset. The antidiuretic hormone arginin-vasopressin is secreted by the neurohypophysis and controls osmotic hemostasis. Copeptin, a relatively novel biomarker, is the c-terminal of the vasopressin precursor hormone and is co-secreted with the hormone [7]. Copeptin has a longer half-life than vasopressin, which makes it easier to detect and it directly reflects vasopressin release. Vasopressin, and thereby copeptin, is released by endogenous stress and increase immediately after chest pain onset [7-9]. The combination of a marker of endogenous stress and a marker of cell necrosis has been suggested to improve the diagnostic performance in chest pain patients at presentation in the emergency department [5,6].

Some publications address the potential benefit of the combination of troponin and copeptin to safely rule out ACS already at presentation [5,6,10-15]. The key findings in these studies are that the addition of copeptin to cardiac troponin seems to allow an early and reliable rule out of AMI. Although suggestive of a potential usefulness of the combination, these studies have either included few patients [10,13,16], used the conventional (4^th^ generation) troponin assays [5,12,16,17], included ST elevation myocardial infarction (STEMI) patients [5,6,10-12] in which biomarkers are of less value, or have only ruled out acute myocardial infarction (AMI) and not the entire ACS population including unstable angina pectoris (UAP) [5,10-12,14,15]. Therefore further studies with sufficient number of patients, using high sensitivity troponin, with clinically relevant endpoints and including UAP patients are needed before copeptin can be safely introduced in routine clinical practise.

The aim of this study was to examine the ability of the combination of copeptin and high sensitivity troponin T (hsTnT) to rule out ACS in patients presenting with chest pain and to compare the diagnostic performance to high sensitivity troponin alone.

## Methods

### Study site

The Skane University Hospital in Lund serves as the primary hospital for approximately 290 000 inhabitants. The hospital has a cardiac intensive care unit with 19 beds and an observation unit with ECG monitoring at 20 beds. Percutanous coronary intervention and coronary artery bypass surgery are available 24 hours a day. The emergency department handles approximately 65000 patient visits per year and 6500 of these present with chest pain. Ambulance ECGs are sent to the cardiologist on call and all STEMI identified are sent directly to the angiography laboratory, bypassing the emergency department.

### Inclusion of patients

During the period of March to July 2011, patients >18 years with chest pain as the primary symptom and who were admitted for observation for suspected ACS were consecutively included. Patients discharged directly after the initial assessment were not enrolled in this study. We excluded patients with STEMI, patients with cardiac arrest during the stay in the emergency department and patients with no available follow up. The study was approved by the regional ethics committee in Lund, Lund university (http://www.epn.se), registry number 2010/429. The regional ethics committee did not request an informed consent.

### Routine clinical assessment and biochemical analysis

All patients underwent routine assessment according to the hospital standards, including physical examination, 12-lead ECG and laboratory analyses including hsTnT. All patients were observed with continuous ECG monitoring, pulse oxymetry and non-invasive blood pressure measurements. Blood samples for hsTnT were collected at admission and thereafter mainly at 3-4 and 5-6 hours from admission according to the hospital routines, or as long as clinically indicated.

In included patients, blood samples drawn at admission were collected and troponin T was determined directly, while copeptin samples were frozen and analyzed later. Copeptin values were analyzed using the BRAHMS copeptin kryptor assay, with a detection limit of 4.8 pmol/L, with a measuring range of 4.8 to 1200 pmol/L and an interassay CV <15% for values <20 pmol/L and an interassay CV <8% for values >50 pmol/L. A copeptin value of <14 pmol/L was used as diagnostic cut-off point in accordance with previous studies [5,6] and the manufacturer’s recommendation. The hsTnT method used was Roche high sensitivity troponin T, with a detection limit of 5 ng/L, with a measuring range of 3-10000 ng/L and an interassay CV of 2%. A troponin T value of ≤14 ng/L was used as diagnostic cut-off point in accordance with current guidelines [1] and the hospital laboratory instructions.

### Data collection and definitions of complications

Variables to calculate GRACE (Global Registry of Acute Cardiac Events) risk score [18] were obtained from hospital records.

Patients who were hypoxic and received treatment with intravenous diuretics or cPAP (continuous positive airway pressure) were classified as having heart failure demanding treatment. A bleeding requiring blood transfusion was classified as a major bleeding. Ventricular fibrillation, ventricular tachycardia and hemodynamic significant bradycardia were defined as malignant arrhythmia.

### Adjudication of the final diagnosis and follow-up

The discharge diagnosis (including the ICD10 code) was made by the responsible specialist ward physician and was obtained from the discharge summary. The final discharge diagnosis was adjudicated by the authors, blinded for copeptin, using all available data. In 5 cases the discharge diagnosis was changed from non-ST elevation myocardial infarction (NSTEMI) to UAP and in 6 cases the diagnosis was changed from UAP to NSTEMI.

The follow-up period was 60 days. A combined primary endpoint of ACS, non-elective percutanous coronary intervention, non-elective coronary artery bypass surgery and death of all causes was used.

The diagnostic criteria for ACS (NSTEMI and UAP) were those recommended by the Swedish national registry for cardiac intensive care (Riks-HIA), which is based on international guidelines from the European Society of Cardiology and the American Heart Association [1,19]. Unstable angina pectoris was defined as typical chest pain history with either STT-changes (T-inversion or ST-depression) or normal ECG, together with normal hsTnT values (≤14 ng/L) or elevated hsTnT values without a significant rising or falling pattern. If the hsTnT concentration was elevated near the cut-off value (between 15-50 ng/L) a 50% change was considered significant. For the diagnosis of UAP a coronary angiography with significant stenosis or a positive stress test was required.

### Statistical analysis

#### Baseline characteristics

Continuous variables are presented as medians with the inter-quartile range and compared with the Mann-Whitney test. Categorical variables are presented as numbers and percentages and compared with the Pearson Chi-square test.

#### Assessment of the diagnostic performance of high sensitivity troponin and copeptin

Biomarkers were treated as categorical variables (positive or negative).

Sensitivity, specificity, negative predictive values (NPV) and positive predictive values (PPV) with 95% confidence intervals were calculated. Sensitivity and specificity were compared with McNemar test or Pearson Chi-square test.

All tests were two-tailed and a p-value <0.05 was regarded as statistically significant.

All statistical analyses were performed with the use of IBM SPSS Statistics 18.

## Results

### Baseline characteristics, outcome and final diagnosis

A total of 493 patients were enrolled in this study and 15 were excluded, 10 with STEMI and 5 with no available follow up. In all 478 patients aged 18-96 years old were included. Baseline characteristics of these patients are shown in Table 1. Patients with ACS were older, had higher values of creatinine and had higher GRACE scores. Furthermore, in the ACS group there was a higher prevalence of male sex, hypertension and hyperlipidemia.

**Table 1:** Baseline characterisctics and presentation in the study population and according to final diagnosis

	**All**	**ACS**	**Not ACS**	**p-value**
	**(n = 478)**	**(n = 107)**	**(n = 371)**	**(ACS vs not ACS)**
***General***				
Age (years)	66 (55-76)	68 (61-78)	65 (54-75)	0.012
Male sex	299 (63)	78 (73)	221 (60)	0.012
***Medical history***				
Hypertension	256 (54)	69 (65)	187 (54%)	0.01
Hyperlipidemia and/or use of statins	185 (39)	54 (51)	131 (35)	0.005
Diabetes	99 (21)	27 (25)	72 (19)	0.19
Smokers	69 (17)	16 (16)	53 (17)	0.84
Ischemic heart disease	184 (39)	47 (44)	137 (37)	0.19
Prior PCI	101 (21)	24 (22)	77 (21)	0.71
Prior CABG	59 (12)	13 (12)	46 (12)	0.95
Stroke/TIA	52 (11)	12 (11)	40 (11)	0.9
Moderate to severe heart failure, EF < 35%	42 (9)	7 (7)	35 (9)	0.35
***On presentation***				
Systolic blood pressure (mmHg)	146 (130-163)	155 (140-172)	143 (130-160)	<0.001
Diastolic blood pressure (mmHg)	80 (75-90)	85 (78-97)	80 (74-90)	0.007
Heart rate (beats/minute)	78 (68-90)	78 (67-89)	77 (69-91)	0.7
ST depression	36 (8)	18 (17)	18 (5)	<0.001
T-wave inversion	40 (8)	17 (16)	23 (6)	0.001
New LBBB	1	0	1	
New RBBB	5	0	5	
CRP (mg/L)	2.1 (0.8-6.7)	2.2 (0.9-4.8)	2.0 (0.76-6.9)	0.87
Creatinine (μg/L)	84 (70-100)	89 (77-107)	82 (69-97)	0.002
Cholesterol (mmol/L)	4.6 (3.7-5.4)	4.6 (3.9-5.5)	4.6 (3.3-5.4)	0.47
GRACE score	104 (82-132)	115 (96-137)	103 (78-130)	0.002
Copeptin (pmol/L)	12 (7-21)	16 (9-30)	12 (7-19)	<0.001
High sensitivity Troponin T (ng/L)	10 (5-25)	25 (13-84)	7 (5-18)	<0.001

*PCI* percutaneus coronary intervention, *CABG* coronary artery bypass grafting, *TIA* transient ischemic attack, *LBBB* left bundle branch block, *RBBB* right bundle branch block, *GRACE* global registry of acute cardiac events. Data presented as n (%) of patients or median and 25th-75th interquartile range for continous variables.

The outcomes for the included patients relative to troponin and copeptin values are shown in Table 2. 107 (22%) patients were diagnosed with ACS during hospital stay, 70 (14%) had NSTEMI and 37 (8%) had UAP. The diagnoses in the 371 patients who did not have ACS were cardiac symptoms from other causes than coronary artery disease in 28% (e.g. atrial fibrillation, stable angina pectoris, congestive heart failure, perimyocarditis), non-cardiac causes in 19% (e.g. musculoskeletal pain, pulmonary embolism, dyspepsia, pneumonia) and chest pain of unknown origin in 53%.

**Table 2:** Complications during study period and outcome related to troponin and copeptin values

	**hsTnT ≤ 14 and Copeptin < 14**	**hsTnT > 14 or Copeptin ≥ 14**	**hsTnT > 14 and Copeptin ≥ 14**	**All**
Total number of patients	n = 204	n = 161	n = 113	n = 478
**Complications**				
Heartfailure demandning treatment	3 (2)	9 (6)	17 (15)	29 (6)
Major bleeding	2 (1)	3 (2)	7 (6)	12 (3)
Malign arrythmia	3 (2)	3 (2)	8 (7)	14 (3)
**Outcome during hospital stay**				
ACS	18 (9)	42 (26)	47 (42)	107 (22)
NSTEMI	3 (2)	30 (19)	37 (33)	70 (14)
Unstable angina pectoris	15 (7)	12 (7)	10 (9)	37 (8)
Coronary angiography with significant stenosis	18 (9)	35 (22)	36 (32)	89 (19)
Coronary angiography without significant stenosis	8 (4)	17 (11)	5 (4)	30 (6)
PCI	11 (5)	23 (14)	27 (24)	61 (13)
CABG	7 (3)	9 (6)	9 (8)	25 (5)
Cardiovascular death	0 (0)	0 (0)	1 (1)	1 (0,2)
Non cardiovascular death	0 (0)	0 (0)	4 (4)	4 (1)
**Outcome after 60 days of follow up**				
ACS during follow up, excluding hospital stay	1 (0,5)	4 (2)	4 (4)	9 (2)
Coronary angiography with significant stenosis during follow up	0 (0)	3 (2)	2 (2)	5 (1)
PCI during follow up	0 (0)	0 (0)	1 (1)	1 (0)
CABG during follow up	0 (0)	3 (2)	0 (0)	3 (0,5)
Death during follow up, excluding hospital stay	0 (0)	0 (0)	4 (4)	4 (1)
Combined endpoint	19 (9)	45 (28)	55 (49)	119 (25)

*ACS* acute coronary syndrome, *NSTEMI* non ST-elevation myocardial infarction, *PCI* percutaneus coronary intervention, *CABG* coronary artery bypass grafting.

Data presented as n (%) of patients. Complications are defined in methods. A combined endpoint of *ACS*, non-elective *PCI*, non-elective *CABG* and death of all causes was used.

Troponin is presentetd in the unit ng/L and copeptin in pmol/L.

5 patients died during the hospital stay and a total of 9 (2%) patients died during the entire study period. All of these patients had elevated troponin values and copeptin values at presentation. The causes of death during the hospital stay were aortic dissection, pulmonary embolism, suspected myocardial infarction and 2 cases of sepsis. The causes of death after discharge were trauma, sepsis and unknown in 2 cases.

### High sensitivity troponin T and copeptin measurements

The diagnostic performance of hsTnT versus the combination of hsTnT and copeptin at admission is presented in Table 3. The sensitivity to detect ACS was significantly increased using the combination of hsTnT and copeptin and this applied to both NSTEMI and UAP. The increased sensitivity resulted in a lowered specificity and the NPV was only slightly increased when using the combination.

**Table 3:** **Sensitivity**, **specificity and predicitve values of high sensitive troponin T and the combination of troponin and copeptin in the final diagnosis of ACS**, **NSTEMI and UAP**

	**hsTnT ≤ 14 ng/L**	**hsTnT ≤ 14 ng/L and Copeptin < 14 pmol/L**	**p-value**
**ACS**			
Sensitivity	69 (59-77)	83 (74-89)	<0.001
Specificity	70 (65-75)	50 (45-55)	<0.001
NPV	89 (84-92)	91 (86-94)	
PPV	40 (33-47)	32 (27-38)	
**NSTEMI**			
Sensitivity	87 (76-93)	96 (86-98)	0.031
Specificity	69 (65-74)	49 (44-54)	<0.001
NPV	97 (94-98)	99 (95-99)	
PPV	32 (26-40)	24 (19-30)	
**UAP**			
Sensitivity	35 (20-52)	59 (42-74)	0.004
Specificity	61 (56-66)	43 (38-48)	<0.001
NPV	92 (88-95)	93 (87-95)	
PPV	7 ( 4-12)	8 (5-12)	

*NPV* negative predicitive value, *PPV* positive predicitive value, *ACS* acute coronary syndrome, *NSTEMI* non ST-elevation myocardial infarction, *UAP* unstable angina pectoris.

Sensitivity, Specificity, *NPV* and *PPV* are given with corresponding 95% confidence interval.

A second hsTnT sample, analysed 3 to 4 hours after the first sample, was available in 309 patients. In Table 4 the comparison in diagnostic performance between hsTnT and copeptin at admission and a repeated hsTnT test after 3-4 hours is shown. Both methods seemed to be equivalent in the NSTEMI subgroup, while the use of the combination resulted in a higher sensitivity in the UAP subgroup. This resulted in a significantly higher sensitivity to identify ACS with the combination of hsTnT and copeptin than with a repeated hsTnT test. Again, this was at the cost of lower specificity.

**Table 4:** **Sensitivity**, **specificity and predicitve values of high sensitive troponin T analyzed 3 to 4 hours after admission and the combination of troponin and copeptin at admission in the final diagnosis of ACS**, **NSTEMI and UAP**

	**hsTnT ≤ 14 ng/L and Copeptin < 14 pmol/L at presentation**	**hsTnT ≤ 14 ng/L after 3-4 hours**	**p-value**
	(**n** = **309**)	(**n** = **309**)	
**ACS**			
Sensitivity	86 (74-92)	77 (65-86)	0.031
Specificity	50 (44-56)	68 (62-74)	<0.001
NPV	92 (85-96)	91 (86-95)	
PPV	33 (26-40)	41 (32-50)	
**NSTEMI**			
Sensitivity	98 (87-100)	98 (87-100)	1
Specificity	49 (43-55)	68 (62-73)	<0.001
NPV	99 (95-100)	99 (96-100)	
PPV	25 (19-32)	35 (27-44)	
**UAP**			
Sensitivity	61 (39-80)	35 (17-57)	0.031
Specificity	42 (37-48)	57 (51-63)	<0.001
NPV	93 (87-97)	92 (86-95)	
PPV	8 (5-13)	6 (3-12)	

*ACS* acute coronary syndrome, *NPV* negative predicitive value, *PPV* positive predicitive value, *NSTEMI* non ST-elevation myocardial infarction, *UAP* unstable angina pectoris.

Sensitivity, Specificity, *NPV* and *PPV* are given with corresponding 95% confidence interval.

Copeptin values related to final diagnosis are shown in Table 5.

**Table 5:** Copeptin values related to final diagnosis

	**Copeptin (pmol/L)**	**p-value (vs not ACS)**
Not ACS	12 (7-19)	
ACS	16 (9-30)	<0.001
NSTEMI	17 (9-44)	<0.001
UAP	14 (9-23)	0.16

Data presented as median and 25th-75th interquartile range.

*ACS* acute coronary syndrome, *NSTEMI* non ST-elevation myocardial infarction, *UAP* unstable angina pectoris.

## Discussion

In this prospective observational study, copeptin in combination with high sensitivity troponin T at admission had a higher sensitivity to identify ACS than hsTnT alone and the use of the combination at admission was found to be equivalent to, or better than, a renewed hsTnT after 3-4 hours. In the NSTEMI subgroup the sensitivity was as high as 98% with a NPV of 99% and this finding is in line with several other studies [5,6,12,14,16,17].

Fewer studies have focused on the entire ACS population. Keller et al. [6] reported a NPV of 80% in the ACS population when combining a 4^th^ generation troponin T test and copeptin. In a relatively small study (n = 58) with high proportion ACS [13] it was reported a sensitivity of 87% and a NPV of 83% when combining hsTnT and copeptin at admission and this was comparable to a second hsTnT sample after 3 hours. These results are similar to our findings.

Although the sensitivity is significantly increased, it is important to note that the NPV increases only slightly, by a few percentages, and this finding is also consistent with other studies [6,10,11,13-15]. In our study of 478 patients, the combination of hsTnT and copeptin decreased the number of false negative tests by 15 as compared to hsTnT alone. This was at a cost of 74 more false positives. Since our study population consisted of only admitted patients and the intention was to identify patients who may be discharged early, we argue that decreasing the amount of false negatives is of importance to maintain an appropriate level of safety.

Overall it seems as if copeptin, a marker of endogenous stress, adds diagnostic information in ACS and that the combination of copeptin and hsTnT is equivalent to, or better than, a second hsTnT value measured 3-4 hours after admission. In the ESC guidelines a rapid rule out of AMI with a second hsTnT after 3 hours is suggested [1]. Our results indicate that hsTnT and copeptin analysed at admission, with a NPV of 99%, can be used to rule out AMI with a very high accuracy, and thus makes a second hsTnT unnecessary.

A clinical problem is the UAP patients, where 24 out of 37 patients in our material were hsTnT- negative at admission. Our study is the first larger study using the combination of hsTnT and copeptin addressing this issue. Reichlin et al. [5] report no significant difference in copeptin concentration between UAP patients and patients with other diagnosis than ACS and attributes this to that UAP does not cause sufficient endogenous stress for vasopressin release. In our material (Table 5), although not statistically significant, the trend was that copeptin values are higher in the UAP patients compared to the non-ACS group, and the addition of copeptin detects 9 of the 24 hsTnT-negative UAP patients. Similar effects when adding copeptin have been reported by other authors [6,13].

If rule out had been based on the combination of hsTnT and copeptin alone, 18 (3 NSTEMI and 15 UAP) out of 107 patients with ACS would have been misdiagnosed as non-ACS. In other words, 9% of the patients who were hsTnT and copeptin negative had a significant stenosis when undergoing a coronary angiography. Most will probably agree that this is too many and we come to the conclusion that biomarkers alone are not sufficient to rule out all ACS. Post-hoc we note that 9 of these patients were identified with stress testing, 7 based on history and ECG alone and 2 due to increasing hsTnT values. As suggested in current guidelines [1] we conclude that history, physical examination, ECG and further evaluation with risk stratification, rule-out of differential diagnosis and possible stress testing is needed. The missed ACS were mainly UAP with negative troponin during the hospital stay. Several previous studies have shown that troponin negative ACS have much better prognosis compared to troponin positive and may not be in need of immediate admission [20]. It could be argued that they could be discharged to later evaluation in the out-patient clinic. For the high-risk group of troponin positive (NSTEMI) patients the NPV at admission was excellent (99%) for the combination missing only 1% of the NSTEMI patients. Since our results indicate that we achieve the same, or better, diagnostic accuracy with the combination of hsTnT and copeptin at admission than with a repeated hsTnT test, we suggest that clinical decisions regarding further investigations (according to guidelines) should be made already in the emergency department, saving at least 3 hours of observation.

Copeptin has also been suggested to be an independent powerful prognostic factor in patients with known coronary artery disease presenting with chest pain [15]. We found that the prognosis was good during 60 days follow-up, with a low frequency of complications and no deaths (Table 2), in the group with negative hsTnT and copeptin. Maybe the combination of hsTnT and copeptin can be used to determine level of care, and allow early discharge in patients without remaining symptoms, with planned follow-up including a stress test when appropriate. This could in our material prevent more than 40% of the admissions of patients presenting with chest pain. It must, however, be kept in mind that the good prognosis may in part be due to that all patients with ACS in this group were intervened during the hospital stay. Further studies where patients are randomized to admission or discharge with follow-up are needed to answer this question, and these studies should also include patients with UAP.

### Study limitations

Firstly, this is a single-center study. However, as our baseline characteristics are comparable to other studies including consecutive patients presenting with chest pain [4-6], we consider our results representative for unselected patient cohorts presenting with chest pain.

Secondly, we have no data on chest pain onset and as previously shown; copeptin is of greatest value early after onset, with an immediate rise, a peak value within 3 hours and a return to baseline in approximately 12 hours [6,9]. The clinical value of copeptin may therefore be greater in early presenters. The pharmacokinetics might also, in patients with low peak values and delay from chest pain onset to blood sampling, cause false negative results. However, in these patients we expect that troponin has had time to rise above the cut off value and in this way the analyses supplement each other.

Thirdly, this is a prospective observational study and therefore we cannot measure the clinical effects of a more sensitive detection of ACS. Intervention studies are needed to provide this important additional information.

## Conclusions

The combination of hsTnT and copeptin analyzed at admission had a higher sensitivity to diagnose ACS than hsTnT alone and the use of the combination at admission was found to be equivalent to, or better than, a renewed hsTnT after 3-4 hours. The combination especially had a strong potential to rule out NSTEMI whereas its capability was weaker in the UAP population. We conclude that biomarkers alone are not sufficient to rule out ACS, but we suggest that the combination of hsTnT and copeptin has the potential to help the clinician in the emergency department to make safe and early decisions on further investigations and on level of care. Furthermore, the use of copeptin in addition to hsTnT may increase the diagnostic accuracy and signal a low risk of complications to an extent where a larger proportion of chest pain patients, in the emergency department, could be further evaluated as outpatients. This could reduce the number of hospitalizations with subsequent lower costs and less inconvenience for the single patient. Future research should address the safety of such an approach.

## Competing interests

Thermo Fisher Scientific Clinical Diagnostics BRAHMS GmbH financed the analysis of copeptin, but had no part in the study design, processing of results or compilation of this research article.

## Authors’ contributions

JT carried out the analysis and interpretation of data, participated in the design of the study and the statistic analysis and drafted the manuscript. CB participated in the analysis and interpretation of data, in the statistic analysis and in the design of the study. DE was involved in drafting the manuscript. BÖ participated in the design of the study and interpretation of data and was involved in drafting the manuscript. All authors read and approved the final manuscript.

## Pre-publication history

The pre-publication history for this paper can be accessed here:

http://www.biomedcentral.com/1471-2261/13/42/prepub
